# Surface-Enhanced Raman Spectroscopy for Bisphenols Detection: Toward a Better Understanding of the Analyte–Nanosystem Interactions

**DOI:** 10.3390/nano11040881

**Published:** 2021-03-30

**Authors:** Eleonora Roschi, Cristina Gellini, Marilena Ricci, Santiago Sanchez-Cortes, Claudia Focardi, Bruno Neri, Juan Carlos Otero, Isabel López-Tocón, Giulietta Smulevich, Maurizio Becucci

**Affiliations:** 1 Dipartimento di Chimica “Ugo Schiff”, Università di Firenze, I-50019 Sesto Fiorentino (Fi), Italy; eleonora.roschi@gmail.com (E.R.); cristina.gellini@unifi.it (C.G.); marilena.ricci@unifi.it (M.R.); 2 Instituto de Estructura de la Materia, IEM-CSIC, Serrano, 121, E-28006 Madrid, Spain; s.sanchez.cortes@csic.es; 3 Istituto Zooprofilattico Sperimentale delle Regioni Lazio e Toscana, I-50010 San Martino alla Palma (FI), Italy; claudia.focardi@izslt.it; 4 Istituto Zooprofilattico Sperimentale delle Regioni Lazio e Toscana, I-00178 Roma, Italy; bruno.neri@izslt.it; 5 Andalucía Tech, Unidad Asociada IEM-CSIC, Departamento de Química Física, Facultad de Ciencias, Universidad de Málaga, E-29071 Málaga, Spain; jc_otero@uma.es (J.C.O.); tocon@uma.es (I.L.-T.); 6 INSTM Research Unit of Firenze, I-50019 Sesto Fiorentino (Fi), Italy; 7 LENS, European Laboratory for Non Linear Spectroscopy, I-50019 Sesto Fiorentino (Fi), Italy

**Keywords:** SERS, cyclodextrin, bisphenol A, bisphenol B, bisphenol S, DFT calculations

## Abstract

Silver nanoparticles functionalized with thiolated β-cyclodextrin (CD-SH) were employed for the detection of bisphenols (BPs) A, B, and S by means of surface-enhanced Raman spectroscopy (SERS). The functionalization of Ag nanoparticles with CD-SH leads to an improvement of the sensitivity of the implemented SERS nanosensor. Using a multivariate analysis of the SERS data, the limit of detection of these compounds was estimated at about 10^−7^ M, in the range of the tens of ppb. Structural analysis of the CD-SH/BP complex was performed by density functional theory (DFT) calculations. Theoretical results allowed the assignment of key structural vibrational bands related to ring breathing motions and the inter-ring vibrations and pointed out an external interaction due to four hydrogen bonds between the hydroxyl groups of BP and CD located at the external top of the CD cone. DFT calculations allowed also checking the interaction energies of the different molecular species on the Ag surface and testing the effect of the presence of CD-SH on the BPs’ affinity. These findings were in agreement with the experimental evidences that there is not an actual inclusion of BP inside the CD cavity. The SERS sensor and the analysis procedure of data based on partial least square regression proposed here were tested in a real sample consisting of the detection of BPs in milk extracts to validate the detection performance of the SERS sensor.

## 1. Introduction

Bisphenols (BPs) are used to improve the quality of plastic materials but have been shown to be endocrine disruptors [1]. Bisphenol A (BPA), the most popular representative of this group, is used for a variety of common consumer goods, such as plastic bottles including water bottles, food storage containers, and sports equipment. Therefore, due to its high production volumes (the global market for plasticizer is about 10 billion US$/year), BPA is considered a “pseudo-persistent” chemical, leading to its spreading and potential accumulation in a variety of environmental matrices, including food, being capable to migrate from the plastic packaging. BPA has been banned in infant feeding bottles across the EU since June 2011 and its use is not allowed in plastic bottles and packaging containing food for babies and children under 3 years since September 2018. The European Food Safety Authority (EFSA) is re-evaluating the risks to public health related to BPA in foodstuffs. This work is expected to conclude in late 2021 or early 2022. The restrictions on BPA usage led manufactories to use alternative BPs, such as bisphenol B (BPB), bisphenol S (BPS), and bisphenol F (BPF). However, these compounds are hazardous substances, and they might have similar toxicity [2,3]. Currently, the Commission Regulation (EU) 2018/213 sets the specific migration limit of BPA from packaging to food to 0.05 mg BPA per kg of food [4]. 

In general, the standard approach for identification and quantification of pollutants is based on sampling, extraction, purification, and chromatographic analysis. It gives very reliable results and it shows a very high sensitivity, but it requires many steps and it is not able to give an immediate feedback on the quality of the samples. SERS spectroscopy has been applied for the detection of different kinds of pollutants due to its sensitivity and specificity [5]. We recently reported a SERS-based early warning detection method of pesticides relevant for food safety and certification of organic products [6,7].

SERS is a very active field of research where new nanomaterials are constantly developed in order to improve the reliability and sensitivity of the experiments, as well as the ease and reproducibility of the measurements. Metal plasmonic nanomaterials sustain localized surface plasmon resonance (LSPR) that leads to an enhancement of the electromagnetic field in their surroundings [8]. Although the fabrication of nanomaterials is crucial in the development of SERS-based sensors, the chemical modification of the metal surface by organic functionalization is an important factor that facilitates the detection of challenging pollutants, such as bisphenols, and provides extra functionality to nanodevices employed in molecular detection [9,10]. In addition, this functionalization can improve the selectivity of the molecular detection by SERS. For instance, the use of Ag and Au nanoparticles decorated with thiolated cyclodextrin (CD-SH) was very useful in the sensing of molecules with hydrophobic core surrounded by hydrophilic groups [11].

Previous SERS-based methods for BPs’ identification were usually of limited sensitivity [12,13] or were presenting highly specific substrates (like metal nanoparticles decorated with molecular imprinted polymers [14,15] or with solid phase microextraction fibers [16] or non-specific, indirect markers (e.g., SERS signals from rapporteur molecules that are modulated by BPs’ presence)) [17,18].

In this work we report the detection of BPs by implementing a SERS nanosensor system where Ag nanoparticles (AgNP) obtained with hydroxylamine [19] functionalized with CD-SH linkers were employed. In addition, density functional theory (DFT) calculations were done to get more insight on the geometry, vibrational assignment of the interacting molecules, and interaction energy occurring in the studied AgNPs-CD-SH-BPs’ systems in order to get a better understanding of the experimental evidences and to stress the key problems that limit the present sensitivity of the method.

## 2. Materials and Methods

### 2.1. AgNP Synthesis and Chemicals

AgNPs were prepared by reducing AgNO_3_ (99.9999% purity, Aldrich, Munich, Germany) with NH_2_OH-HCl (99.9% purity, Aldrich) in extra-pure distilled water (HPLC grade, Lichrosolv, Merck, Kenilworth, NJ, USA) according to Leopold and Lendl’s procedure [19]. The silver nanoparticles were remarkably stable, conserving their distinctive surface plasmon band centered at 410 nm for several weeks (see Figure S1, black line).

The BP samples were obtained from Sigma-Aldrich (99.99% purity) and prepared as 100 ppm (~4 × 10^−4^M) methanol stock solutions.

The 6-deoxy-6-thio-β-cyclodextrin (CD-SH) used for AgNPs’ functionalization was provided by Zhiyuan Biotechnology (Binzhou, China).

### 2.2. Instrumentation

Extinction measurements were obtained in a quartz cuvette, 2-mm optical pathlength, by using an UV-Vis-NIR Agilent Cary60 spectrometer (Agilent Technologies, Santa Clara, CA, USA).

MicroRaman measurements were performed by means of a Renishaw 2000 spectrometer equipped with the 785-nm line from a diode laser, incident power over 3 mW, coupled with a Leica DLML confocal microscope with 60x objective, two notch filters, and a single grating (1200 lines mm^−1^) monochromator. The spectrometer was routinely calibrated with respect to the 520-cm^‒1^ band of a silicon wafer. The Raman and SERS spectra were calibrated with indene as standards to an accuracy of 1 cm^−1^ for intense isolated bands. The back-scattered Raman signal was collected and focused into the monochromator through 40-μm slits and detected by a Peltier-cooled CCD multichannel detector operating at −20 °C.

### 2.3. SERS Measurements

The reference samples employed for the SERS experiments focused on method development were prepared by functionalization of AgNPs (100 μL of solution) with 6-deoxy-6-thio-β-cyclodextrin (CD-SH, 50 μL of a 10^−4^ M water solution). The time-dependent chemiadsorption of CD-SH on AgNPs was monitored by UV-Vis spectra measurements. The red line in Figure S1 shows the UV-Vis spectrum of functionalized AgNPs measured 30 min after mixing. Only a minor red shift (2 nm) and a small intensity decrease due to dilution were observed. However, no evidence of aggregation was found in the longer wavelength region. One droplet of this solution was deposited on a clean, glass microscope slide, dried in air at room temperature, and used as reference sample in SERS measurements. Then, a few μL of the BPs’ standard solutions were added to the AgNPs/CD-SH solution to reach the desired concentration, ranging from 2.5 × 10^−5^ to 8 × 10^−7^ M, and incubated for 20 min. The higher concentration value was limited by the CD-SH final concentration in solution (~3 × 10^−5^ M). The relative extinction spectrum did not show any further change. One droplet of the resulting solution was then deposited on a clean, glass microscope slide and dried in air at room temperature. The SERS measurements were performed on the external corona of the droplet where the AgNP density was higher. Each SERS spectrum was obtained with 0.3-mW laser power acquiring 10 scans for 10 s each. The observed SERS spectra were very reproducible. Therefore, the final spectra were obtained by adding 30 spectra taken from different positions of the drop and averaged. Each final spectrum was baseline corrected.

The developed method was preliminarily tested on milk samples spiked with known amounts of BPA. These samples were prepared in the laboratory starting from commercial milk and BPA stock solutions in methanol. Milk is a complex matrix for SERS analysis [20]. Therefore, we adopted a recently proposed method for BPA extraction from milk [21]. Molecular imprinting polymers (MIP) were used as sorbents for BPA pre-concentration in milk samples. Spiked samples at the nominal concentration of 3 mg/kg were prepared, adding 0.15 mL of BPA stock solution (1 mg/mL) to 50 g of homogenized milk, previously tested by standard chromatographic methods to demonstrate the absence of BPA residues, under a gently magnetic stirring. Then, the sample was continuously stirred for 15 min to ensure a good level of homogeneity. After homogenization, 10 g of samples were centrifuged for 25 min at 3500 rpm to eliminate fat components. Skimmed milk was passed through the MIP column Affinimip-SPE (Affinisep, F). The column was washed with 6 mL of a water/acetonitrile mixture (60/40, *v*/*v*) and, finally, BPA was eluted with 6 mL of methanol. A limited amount of this solution (10 μL) was added to the 150-μL mixed colloid/CD-SH solution described above. After mixing and 20 min equilibration, a droplet of this solution was used for SERS measurements, following the procedure already described.

### 2.4. Multivariate Analysis of SERS Data

We analyzed the measured SERS spectra of the samples with different BPs’ concentrations by multivariate statistical analysis in order to validate the possible quantitative determination of the analytes [22].

The calibrated spectra were regularly resampled at 2-cm^−1^ step in the spectral region 410–1390 cm^−1^. Smoothing was performed using the Savitzky–Golay method second-order polynomial on seven data points). In order to carry out the data analysis, a data normalization procedure was mandatory to make comparable the different sets of data associated to the experimental procedure. We tested both normalizing the spectra on the CD-SH strong band at 480 cm^−1^ or by applying the standard normal variate (SNV) transformation. The results on reference materials were not dependent on the choice of the normalization procedure.

### 2.5. Theoretical Calculations

B3LYP [23,24] density functional theory (DFT) functional as implemented in Gaussian16 [25] suit of programs was selected for electronic structure calculations of geometries, vibrational wavenumbers, and interaction energies of BPs in the discussed systems. The 6–31G* [26,27] as well as the LanL2DZ [28,29,30] core potential were used as basis sets, although both of them provided very similar results. Steric hindrance between the alkyl substituents of the central carbon atom and the two aromatic rings of BPs led to a distorted geometry with C_2_ symmetry, which was 14.5 kcal/mol more stable than the C_2v_ symmetry structure of BPA, for instance (B3LYP/6-31G*, Figure S3a), while the optimized geometry of BPS could be considered as C_2v_ (Figure S4a). Interaction energies ΔE_A…B_ between two A and B systems were estimated as usual, ΔE_A…B_ = E_AB_ − E_A_ − E_B_, and could be only considered as estimative, given they were small and the solvent was not taken into account in the calculations. Simple approaches of solvent effects using, for instance, polarizable continuum model (PCM) were unable to account for the hydration of these complex systems [31]. Discussion was focused on BPA derivative, given that all BPs had very similar calculated vibrational wavenumbers and interactions’ energies.

## 3. Results and Discussion

### 3.1. Raman and SERS Spectroscopy Results

**Figure 1:**
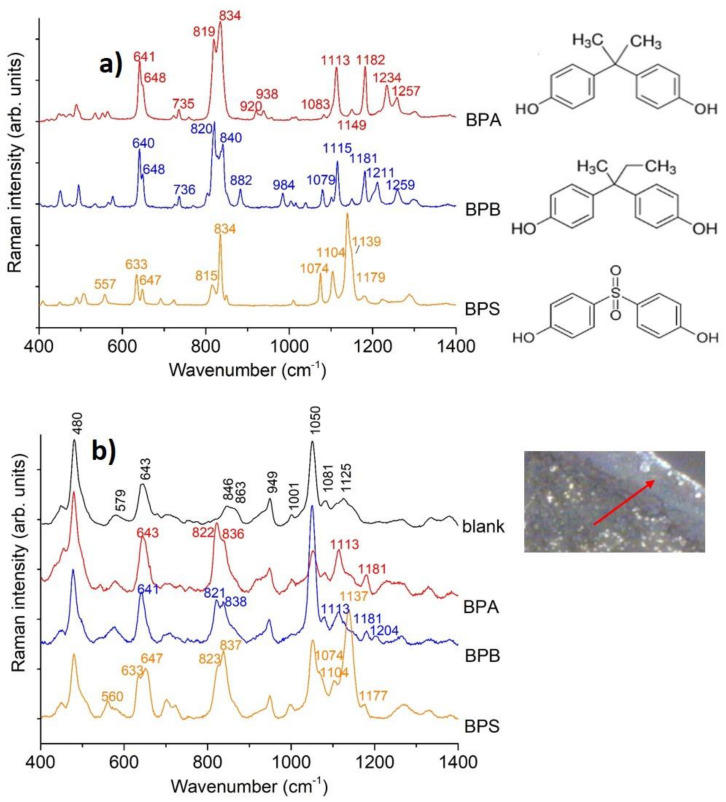
(**a**) Raman spectra of solid BPs (laser power 1 mW, exposure 50 s, 5 accumulations). (**b**) SERS spectra measured on dried droplets with BPs and AgNPs-SH-CD (analyte concentration 2.5 × 10^−5^ M). The arrow in the inset shows the droplet region where the measure was performed (photo taken with the 60× objective and instrument camera). Raman intensities were normalized with respect to the 480 cm^−1^ band of CD-SH (laser power 0.3 mW).

The Raman spectra of the different BPs’ solid samples together with their chemical structures are shown in Figure 1a. Some characteristic bands can be found common to all BPs, whose assignments were previously reported [32,33] (Table S1) and were reassigned according to B3LYP/LanL2DZ force field results (see below, Table 1 and Table S2).

**Figure 2:**
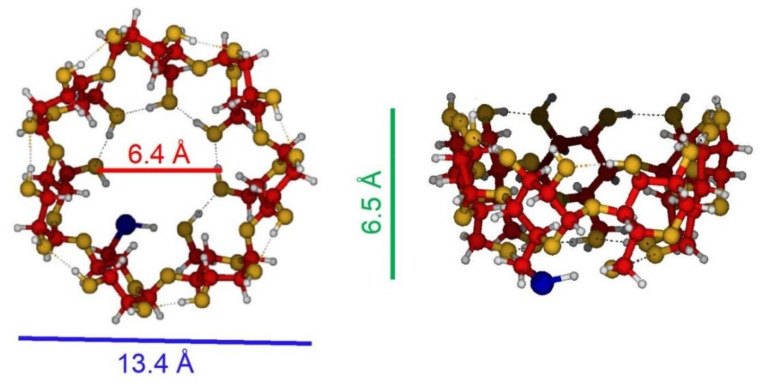
B3LYP/LanL2DZ optimized geometry of 6-deoxy-6-thio-β-cyclodextrin (CD-SH).

In order to obtain good quality SERS spectra, AgNPs were selected as the active substrate because these systems have a higher LSPR performance and the plasmon resonance of silver is much larger and encompasses a longer region of the electromagnetic spectrum with respect to the case of other metal (e.g., gold or copper) nanoparticles. The first attempts to observe the BPs’ SERS spectrum in a liquid sample, simply by mixing the AgNPs’ colloidal dispersion to a BPs’ solution, failed. This result was expected since it is long time that the low affinity of BPs toward metallic surfaces, like that of Au and Ag nanostructures, is known [12,34,35,36]. To circumvent this limitation, the experimental method was designed to improve the BPs–AgNP interaction and, therefore, SERS detection. The method of choice was a functionalization of the AgNPs with a suitable molecule acting as a cross-linker. Among all the possible molecular systems to be employed, such as pyridine [36], calixarenes [37,38], or graphene [39], we focused on β-cyclodextrin (CD) [40,41]. In particular, we used those modified with a thiol group 6-deoxy-6-thio-β-cyclodextrin (CD-SH, see Figure 2), since the presence of this group provides a strong interaction with the silver substrate and the molecular structure, and dimensions of the hydrophobic oligosaccharide cavity could allow for interaction with guest molecules with smaller size than that of the cavity [40]. However, experiments performed on the colloidal solution containing the functionalized metal nanoparticles (AgNPs-SH-CD) and BPs at concentration up to 10^−4^ M did not give rise to any SERS signal of the analytes. Only measurements on samples prepared by dropcast methods using AgNPs-SH-CD as the active SERS substrate provided a satisfactory sensitivity for BPs’ detection in low concentrations.

The BPs’ SERS spectra we obtained were quite similar to the corresponding Raman spectra (Figure 1a,b and Figure S2). For instance, in the case of BPA, the SERS spectrum was characterized by the five major peaks at 643, 822, 836, 1113, and 1181 cm^−1^. Most of these vibrational frequencies were not shifted with respect to the Raman bands; the only exception is represented by the doublet around 820–840 cm^−1^, which moved 4–6 cm^−1^ toward higher frequencies. Moreover, for the BPA case, an inversion of the relative intensity of these two strong bands between Raman and SERS was clearly observed.

### 3.2. DFT Calculations

In order to gain some insight on the properties of these systems, we performed density functional theory (DFT) calculations on the geometry, vibrational wavenumbers, and interaction energy occurring in the studied AgNPs-SH-CD-BPs’ systems. According to previous results [42,43,44], we used a neutral, closed shell, Ag_2_ dimer as a simple model of the AgNPs in order to evaluate the affinity of BPs or CD for the nanoparticles. Other options, like a single Ag^+^ ion, were previously used in this kind of calculation, but this cationic species had a too high positive excess of charge and would correspond to an ionized atom, which was not part of the metal (nanoparticle or electrode) structure [44]. Anionic charged species (Ag^−1^) was also discarded because neutral molecules are preferably adsorbed on neutral or positive charged locations of the metal surface.

The first set of calculations elucidated the stability of the BPs-AgNPs’ adducts in water solution. B3LYP/LanL2DZ results indicated that not significant differences were expected between the adsorption energies of BPA and BPB or BPS on Ag_2_ (as reported below) and, therefore, only those relative to BPA will be discussed. The interaction between Ag_2_ and BPA occurred through a hydroxyl group and the calculated stabilization energy amounted to ΔE_Ag2…BPA_ = −7.76 Kcal/mol (see Figure S3b). This interaction energy was smaller than that calculated for the respective complexes with a water molecule (ΔE_Ag2…H20_ = −11.70 and ΔE_H20…BPA_ = −11.74 Kcal/mol, respectively, Figure S3c,d). This result suggests that AgNPs and BPA prefer to be hydrated rather than forming a complex between them and also explains why BPs were unable to aggregate silver sols, which prevented recording their SERS spectra in aqueous solution.

The role of the thiol group of CD-SH in the functionalization of AgNPs was also evaluated. It is well known that thiols are adsorbed on noble metal surfaces as thiolates [45]. B3LYP/LanL2DZ results confirmed this kind of interaction, given that CD-SH deprotonates in the optimization process of the CD-SH…Ag_2_ complex. The -SH thiolic proton migrated toward a close hydroxyl group, giving the H_2_O^+^-CD-S^−^…Ag_2_ species (ΔE_H2O+-CD-S…Ag2_ = −15.98 Kcal/mol, Figure S3e).

**Figure 3:**
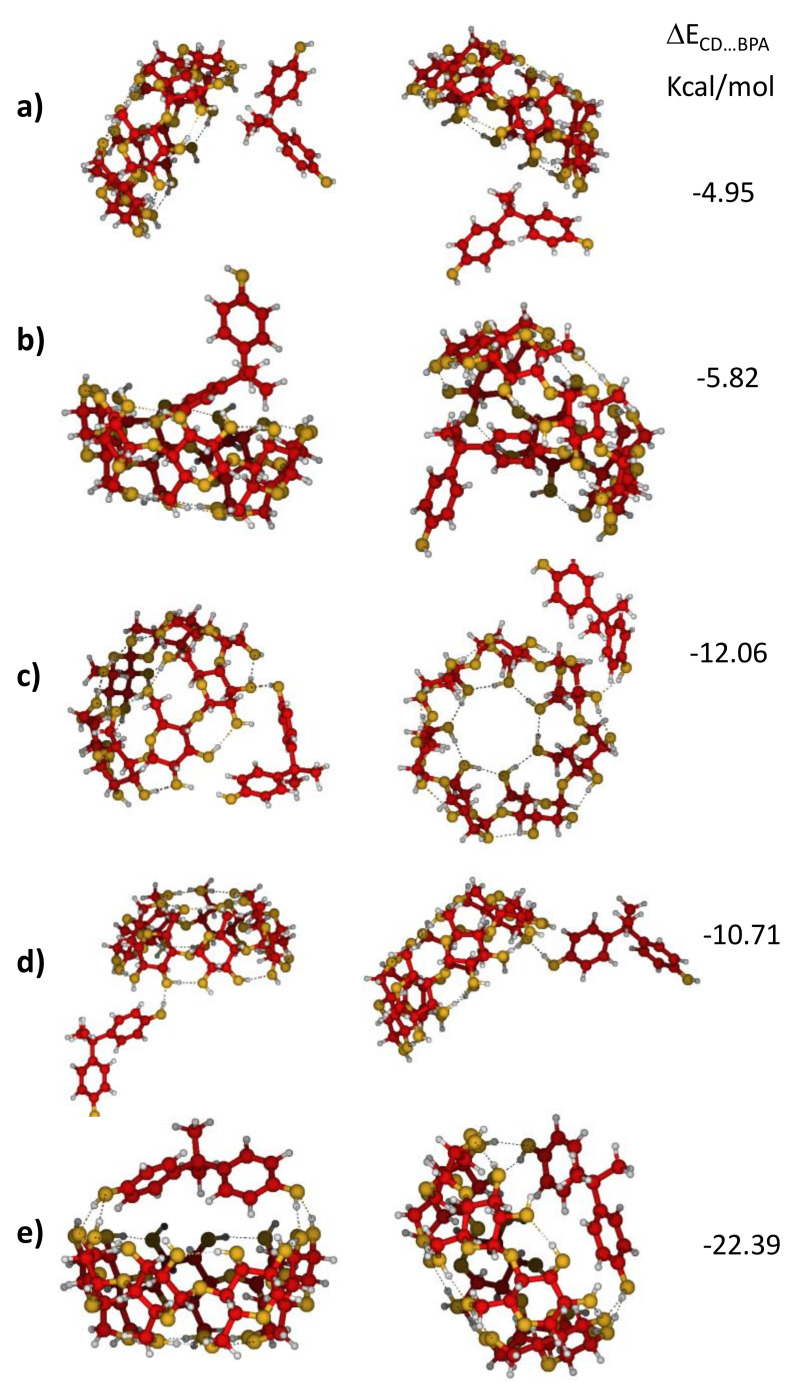
B3LYP/6-31G* optimized geometries and interaction energies of different complexes formed between BPA and CD. The nature of interaction in geometries (**a**,**b**) is hydrophobic, in geometries (**c**,**d**) is single hydrogen bond, and in geometry (**e**) is double hydrogen bond.

**Figure 4:**
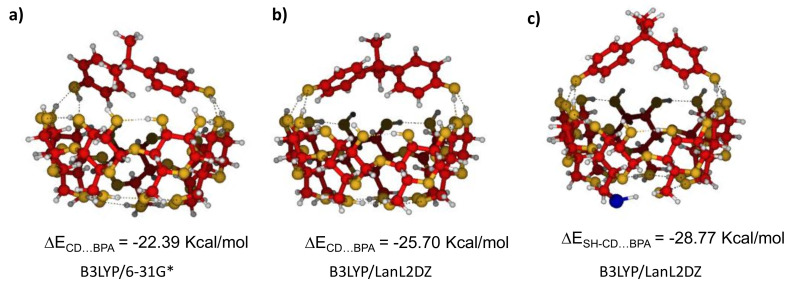
Optimized geometries and calculated interaction energies of the complex formed between BPA and CD (**a**,**b**) or SH-CD (**c**).

CDs and BPs can be considered as both hydrophobic and hydrophilic species because they have alkyl and aromatic parts as well as several -OH polar groups able to bond to water molecules through hydrogen bonds. Some of them should be broken when the CD…BPs’ complex is formed, and this loss of energy must be compensated with the stabilizing ΔE_CD…BP_ energy of the new interaction. Geometry optimization gave different final structures of the CD…BPA supramolecular complexes, which can be classified in three kinds, according to the nature of the interaction: (1) hydrophobic (Figure 3a,b), (2) single hydrogen bond (Figure 3c,d), and (3) double hydrogen bond (Figure 3e). The corresponding B3LYP/6-31G* calculated energies were around of −5, −10, and −25 kcal/mol, respectively, which means that CD…BPA interactions were not too strong, being dominated for a face-to-face chelating coordination, which was stabilized by several hydrogen bonds between hydroxyl groups of CD and BPA (see Figure 3a–e). The geometry and energy of this preferred complex CD…BPA was similar to other BPs and did not depend very much on the level of calculation or whether BPA was bonded to CD or SH-CD (Figure 4 and Figure S4). However, −25 kcal/mol energy of stabilization may be not sufficient to preserve the complex upon solvation where several hydrogen bonds can be formed between water and hydroxyls of both moieties, CD and BP. This is the most plausible explanation for the failure to record SERS signals of BPs in aqueous samples.

The vibrational spectrum of BPA, as isolated molecule or bound to CDs, was calculated as well. The most relevant bands of the Raman spectrum are reported in Table 1. Table S2 summarizes the comparison between experimental and calculated wavenumbers of the characteristic Raman bands of the three BPs under study.

Our calculations agreed with the results published by Ullah and Zheng [32,33], but the assignment of particular bands was modified in view of the SERS results where in-plane vibrations of benzene-like derivatives played a main role. BPs have many vibrations with complex motions and some of them are calculated at very similar wavenumbers. For instance, five fundamentals were calculated between 800 and 825 cm^−1^ while only two strong Raman/SERS bands were recorded, which means that the final assignment could contain some grade of uncertainty. In particular, (1) the intense experimental band around 640 cm^−1^ was assigned to the 6b normal mode and corresponded to the in-plane deformation of the aromatic ring together with a contribution of a stretching of the CCC bonds between the two aromatic rings; (2) the two strong peaks in the region 820–840 cm^−1^ were assigned to in-plane ring-breathing vibrations of the rings; and (3) the bands around 1100–1200 cm^−1^ were mainly due to deformations of aromatic CH bonds, with contribution from the CC stretching coordinates. At higher wavenumbers, vibrational contributions deriving from CO and CC stretching bonds were found.

Atomic displacement of the main vibrations can be seen in Figure S5.

SERS and Raman spectra were rather similar since BPA was not directly bonded to the silver nanoparticle, as our calculations indicated that it was bound to CD-SH only through the hydroxyl group, as shown in Figure 3 and Figure 4, and Figure S4. The only exception shown by our experimental data is represented by the two strongest bands around 820 and 840 cm^−1^, which moved 2–4 cm^−1^ toward higher wavenumbers and inverted their relative intensity between Raman and SERS (see Figure 1 and Table 1). These bands are characteristic vibrations of p-substituted benzene derivatives and correspond to Wilson´s mode 1, νring, an in-plane deformation of the benzenic ring [46]. The behavior of the intensities of these bands and the small wavenumber shifts could have been due to the loss of equivalence between the two aromatic rings under adsorption.

Figure S4 shows the optimized geometries and the interaction energies of the complexes formed by the different BPs with CD and CD-SH. The similarity between them explains why all the SERS of BPs look similar and the weak energies of the complexes account for the resemblance of the respective Raman and SERS wavenumbers and intensities.

### 3.3. Multivariate Analysis of SERS Data

**Figure 5:**
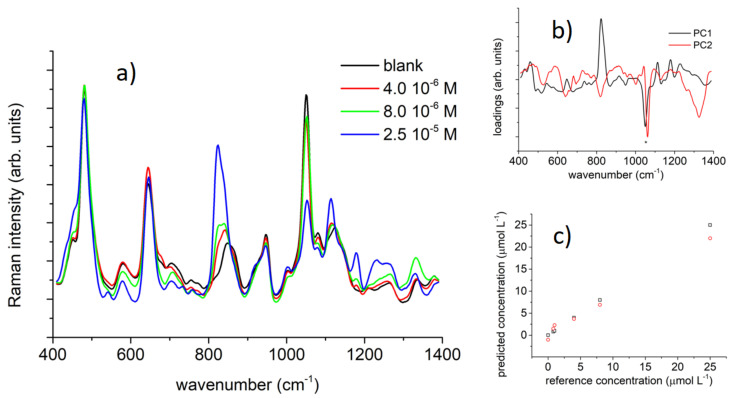
Relevant SERS data for BPA and the multivariate analysis. (**a**) BPA SERS spectra as a function of the nominal BPA molar concentration (SNV data transformation and smoothing applied). (**b**) Loadings for the first two components in the PCA analysis on the BPA SERS data (full spectrum). PC1 and PC2 were carrying the 82 and 9% of the spectral variance, respectively. (**c**) Predicted (black square) and validated (red circle) concentration of BPA in model samples vs. reference analytical concentration as obtained by the partial least square (PLS) regression analysis (4 factors model) on SERS data (full spectrum).

The data were evaluated with principal components analysis (PCA) and the correlation between the spectral data and the analyte concentration was studied with partial least square (PLS) regression. We report in Figure 5 the results (experimental spectra, loadings of the PCs, and calibration/validation plots) obtained on BPA, as a representative case for this study. Data on BPB and BPS are available in the Figures S6 and S7. The loadings of the first two principal components, PC1 and PC2, were closely related to both the analyte and CD-SH spectrum and showed all the major relevant peaks of both reference Raman spectra (see BPA results in Figure 5a,b). Furthermore, the analysis was carried out on the full spectral range or focusing on small regions where the BPs’ bands were stronger and growing on a flat background. The results were quite similar. However, we preferred to work in the spectral region around the main peaks at 820–840 cm^−1^ that showed the more evident changes with BPs’ concentration. This could be more important working on real samples where some bands in the background signal could affect the calibration carried out on model samples. 

The PLS models built on BPA, BPB, and BPS were quite satisfactory. The plot of the calibration vs. reference data was linear (Figure 5c, Figure S6c and Figure S7c, R-square > 0.999). The root mean square (RMS) error in the calibration process was equal or below 10^−7^ M (1.0 × 10^−8^ M for BPA, 2 × 10^−8^ M for BPB, and 8 × 10^−8^ M for BPS). These values were very similar for the three BPs because they only differed in the central sp^3^ group, which did not interact directly with CD-SH. However, the slightly higher value (i.e., the lower sensitivity of the method) found in the case of the more polar BPS could be due to the presence of the polar −SO_2_ group, which favors the water solubility, diminishing its concentration at the interface.

The RMS error in the validation process carried out with the leave-one-out cross-validation method was quite similar for the different BPs we studied (~3 × 10^−7^, limiting the evaluation to the lower concentration range). Out of these numbers, we assumed that in our SERS experiments the limits of detection and quantification for BPs by the SERS method we presented were approximately 3 × 10^−7^ M and 1 × 10^−6^ M, respectively.

**Figure 6:**
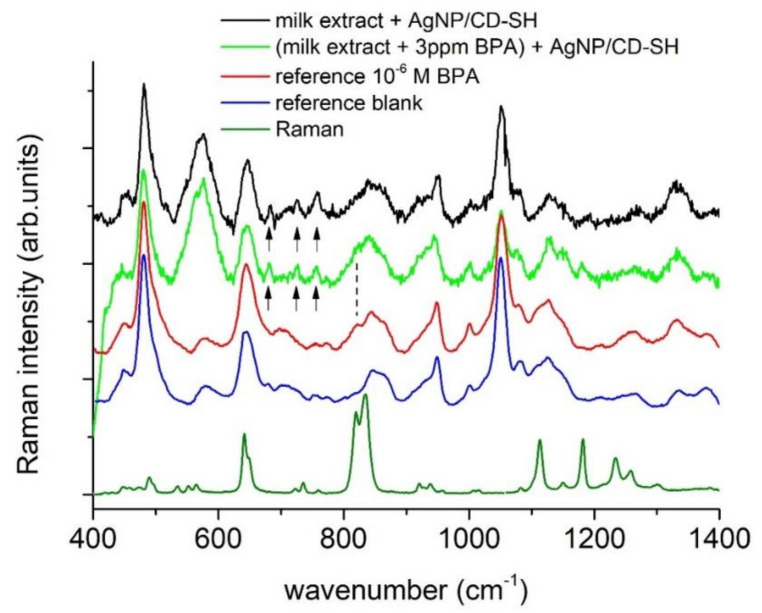
SERS spectra of both 1.0 × 10^−6^ M BPA samples and corresponding blanks from reference materials (bottom traces) and milk extracts (BPA spiked and blank). The dotted line indicates the BPA relevant band at 822 cm^−1^ and the arrows indicate the spurious bands from milk.

Furthermore, we made preliminary tests trying to verify the application of this approach for determination of BPs in milk extracts. Milk extracts in methanol, obtained by the SPE (solid phase extraction) method, showed some feeble opalescence. The SERS spectrum of the milk blank (see Figure 6) showed some limited new spectral features with respect to the reference material, associated to the milk matrix, e.g., the weak peaks at 677, 723, and 751 cm^−1^ (marked with a small arrow). The changes in the spectrum induced by the BPA contamination (marked with a vertical line) were remarkably similar in the two samples (milk extracts and test materials) containing the same quantity of analyte (1 × 10^−6^ M in the final solution). This suggests the possible application of SERS in the determination of BPs in milk extracts.

## 4. Conclusions

Basic SERS methods allow for a very limited sensitivity in BPs’ SERS detection, in the order of 10^−3^ M [12]. We improved by about 3 orders of magnitude the detection of BPs by SERS spectroscopy, developing simple and repeatable SERS active substrates thanks to the knowledge of the substrate/molecule/solvent interactions. These interactions were modeled by means of DFT calculations on simple reference models. We used a conventional silver colloid, prepared by the well-known Leopold and Lendl method [19], and we showed that the use of a cross-linker, such as a commercial β-cyclodextrin modified with a thiol group, improved the AgNP-BPs’ interaction and, therefore, the SERS spectrum in dry conditions. However, the BPs-SERS substrate interaction energy was still quite small and the interaction with the solvent water molecules strongly limited the BPs’ SERS detection in solution, even using AgNPs functionalized with cyclodextrin. Dry samples, prepared by dropcast method, allowed for BPs’ detection with good sensitivity and repeatability in standard and realistic samples. The data analysis required some standard multivariate methods due to the partial overlap of relevant bands of BPs and cyclodextrin and the selection of specific spectral ranges due to the presence of other signals from the milk matrix. However, even in real samples it is possible to obtain the detection of BPs’ presence and possibly their quantification, thanks to an accurate calibration procedure. It is quite evident that the weak interactions occurring in the system AgNPs-CD-BPs that were responsible for its limited stability require a thorough sample preparation, e.g., by SPE methods, in order to avoid interferences that possibly can occur working on real samples, such as milk or other dairy food.

Further direction for an improvement of the method sensitivity, while retaining its ease and generality of application, could be the identification/development of a more specific linker between AgNPs and BPs able to bind more tightly and selectively to the analyte.

Another remark concerns the multiplexing detection of different BPs present in a sample. The method we present is able to detect with comparable sensitivity BPA, BPB, and BPS. Our evidences suggest that differentiation between BPA and BPB is not possible: Their Raman spectra show only minor differences that, in SERS, are completely concealed by the observed line broadening. Instead, BPS in a mixture can be identified, thanks to the presence of the specific SERS band at 1137 cm^−1^ involving the S-O stretching vibration.

## Figures and Tables

**Table 1:** Assignment of the experimental Raman/SERS spectra and calculated vibrational wavenumbers (cm^−1^) of BPA and its complexes with CD and CD-SH.

Experimental		B3LYP/6-31G *	B3LYP/LanL2DZ			This Work	Refs. [32,33]
Raman	SERS	BPA	BPA	SH-CD-BPA ^b^	CD-BPA ^b^	Assignment ^a^	Assignment ^c^
489	495 ^1^	543	542	548	549	δCCCskeletal	γ(CO)
641	643 ^2^	645	638	644	642	6b; δring + νCring-C-Cring	δ(CCC)r
648		657	657	658	658	6a; δring	ν(CC)
735	735	744	752	756	754	4; τring	τ(ring), γ(CO)
819	822	847	828	832	830	1; νring	γ(CH), τ(HCCC)
834	836	857	839	842	841	1; νring + νCring-C-Cring	δ(CCC)r, γ(CH)
920	920	950	937	938	927	rCH_3_ + νCCH_3_	ν(CC)
938	938	958	955	954	951	rCH_3_ + νCCH_3_	ν(CC), ω(CCH)r
1083		1110	1109	1112	1110	δCHring + νCCring + rCH_3_	ν(CC)
1113	1113	1133	1136	1139	1136	δCHring + νCCring	ν(CC), ω(CCH)r
1149		1178	1175	1186	1179	δCHring + νCCH_3_	ν(CC)
1182	1181	1210	1218	1215	1215	δCOH + CHring	ν(CC), ω(CCH)r
1234		1268	1258	1276	1279	νCring-C-Cring + rCCH_3_	ν(CO)
1257		1308	1274	1294	1310	νCO + νCCring + δCHring	ν(CC)

^1^ Overlapped with the band at 480 cm^−1^ of CD-SH; ^2^ overlapped with the band at 643 cm^−1^ of CD-SH; ^a^ ν: stretching; δ: in-plane deformation; r: rocking; τ: torsional deformation. Wilson’s nomenclature for the benzene-like normal modes, 6a, 6b, 1, and 4 [46]. Vibrational modes visualized by using MOLDEN program [47]; ^b^ vibrational wavenumbers of the most stable CD-BPA complex (see Figure 3e); ^c^ ω, wagging; r, ring.

## Data Availability

Data are available from the corresponding Authors.

## Supplementary Materials

The following materials are available online at https://www.mdpi.com/article/10.3390/nano11040881/s1. Tables S1 and S2: Vibrational assignment of BPA, BPB, and BPS. Figure S1: Localized surface plasmon resonance (LSPR) band of silver nanoparticles from synthesis and after functionalization with CD-SH. Figure S2: The background-subtracted SERS spectra of the different BPs compared to the corresponding Raman spectra. Figure S3: Optimized geometries of BPA and interaction energies of different complexes formed between BPA, Ag_2_, H_2_O, and CD-SH. Figure S4: Optimized geometries and calculated energies of isolated BPs and of the complexes formed between BPs and CD. Figure S5: Atomic displacement of the main vibrational normal modes of BPA. Figure S6: Multivariate analysis on the BPB SERS data. Figure S7: Multivariate analysis on the BPS SERS data.

## References

[1] Maffini M.V., Rubin B.S., Sonnenschein C., Soto A.M. (2006). Endocrine disruptors and reproductive health: The case of bisphenol-A. Mol. Cell. Endocrinol..

[2] Eladak S., Grisin T., Moison D., Guerquin M.J., N’Tumba-Byn T., Pozzi-Gaudin S., Benachi A., Livera G., Rouiller-Fabre V., Habert R. (2015). A new chapter in the bisphenol a story: Bisphenol S and bisphenol F are not safe alternatives to this compound. Fertil. Steril..

[3] Cano-Nicolau J., Vaillant C., Pellegrini E., Charlier T.D., Kah O., Coumailleau P. (2016). Estrogenic effects of several BPA analogs in the developing zebrafish brain. Front. Neurosci..

[4] EUR-Lex—32018R0213—EN—EUR-Lex. https://eur-lex.europa.eu/eli/reg/2018/213/oj.

[5] Pang S., Yang T., He L. (2016). Review of surface enhanced Raman spectroscopic (SERS) detection of synthetic chemical pesticides. TrAC Trends Anal. Chem..

[6] Tognaccini L., Ricci M., Gellini C., Feis A., Smulevich G., Becucci M. (2019). Surface Enhanced Raman Spectroscopy for In-Field Detection of Pesticides: A Test on Dimethoate Residues in Water and on Olive Leaves. Molecules.

[7] Feis A., Gellini C., Ricci M., Tognaccini L., Becucci M., Smulevich G. (2020). Surface-enhanced Raman scattering of glyphosate on dispersed silver nanoparticles: A reinterpretation based on model molecules. Vib. Spectrosc..

[8] Aroca R. (2007). Surface-Enhanced Vibrational Spectroscopy.

[9] Perumal J., Wang Y., Attia A.B.E., Dinish U.S., Olivo M. (2021). Towards a point-of-care SERS sensor for biomedical and agri-food analysis applications: A review of recent advancements. Nanoscale.

[10] Cupil-Garcia V., Strobbia P., Crawford B.M., Wang H., Ngo H., Liu Y., Vo-Dinh T. (2021). Plasmonic nanoplatforms: From surface-enhanced Raman scattering sensing to biomedical applications. J. Raman Spectrosc..

[11] Koh E.H., Moon J.Y., Kim S.Y., Lee W.C., Park S.G., Kim D.H., Jung H.S. (2021). A cyclodextrin-decorated plasmonic gold nanosatellite substrate for selective detection of bipyridylium pesticides. Analyst.

[12] Bakar N.A., Salleh M.M., Umar A.A., Shapter J.G. (2017). Design and measurement technique of surface-enhanced Raman scattering for detection of bisphenol A. Adv. Nat. Sci. Nanosci. Nanotechnol..

[13] Lin P.Y., Hsieh C.W., Hsieh S. (2017). Rapid and Sensitive SERS Detection of Bisphenol A Using Self-assembled Graphitic Substrates. Sci. Rep..

[14] Ren X., Cheshari E.C., Qi J., Li X. (2018). Silver microspheres coated with a molecularly imprinted polymer as a SERS substrate for sensitive detection of bisphenol A. Microchim. Acta.

[15] Xue J.Q., Li D.W., Qu L.L., Long Y.T. (2013). Surface-imprinted core-shell Au nanoparticles for selective detection of bisphenol A based on surface-enhanced Raman scattering. Anal. Chim. Acta.

[16] Qiu L., Liu Q., Zeng X., Liu Q., Hou X., Tian Y., Wu L. (2018). Sensitive detection of bisphenol A by coupling solid phase microextraction based on monolayer graphene-coated Ag nanoparticles on Si fibers to surface enhanced Raman spectroscopy. Talanta.

[17] Feng J., Xu L., Cui G., Wu X., Ma W., Kuang H., Xu C. (2016). Building SERS-active heteroassemblies for ultrasensitive Bisphenol A detection. Biosens. Bioelectron..

[18] Lin L.K., Stanciu L.A. (2018). Bisphenol A detection using gold nanostars in a SERS improved lateral flow immunochromatographic assay. Sens. Actuators B Chem..

[19] Leopold N., Lendl B. (2003). A new method for fast preparation of highly surface-enhanced raman scattering (SERS) active silver colloids at room temperature by reduction of silver nitrate with hydroxylamine hydrochloride. J. Phys. Chem. B.

[20] Marques A., Veigas B., Araújo A., Pagará B., Baptista P.V., Águas H., Martins R., Fortunato E. (2019). Paper-Based SERS Platform for One-Step Screening of Tetracycline in Milk. Sci. Rep..

[21] Kang J.H., Kondo F. (2003). Determination of bisphenol A in milk and dairy products by high-performance liquid chromatography with fluorescence detection. J. Food Prot..

[22] Martens H., Karstang T., Næs T. (1987). Improved selectivity in spectroscopy by multivariate calibration. J. Chemom..

[23] Becke A.D. (1993). Density-functional thermochemistry. III. The role of exact exchange. J. Chem. Phys..

[24] Stephens P.J., Devlin F.J., Chabalowski C.F., Frisch M.J. (1994). Ab Initio calculation of vibrational absorption and circular dichroism spectra using density functional force fields. J. Phys. Chem..

[25] Frisch M.J., Trucks G.W., Schlegel H.B., Scuseria G.E., Robb M.A., Cheeseman J.R., Scalmani G., Barone V., Petersson G.A., Nakatsuji H. (2016). Gaussian 16, Revision C.01. https://gaussian.com/citation/.

[26] Petersson G.A., Bennett A., Tensfeldt T.G., Al-Laham M.A., Shirley W.A., Mantzaris J. (1988). A complete basis set model chemistry. I. The total energies of closed-shell atoms and hydrides of the first-row elements. J. Chem. Phys..

[27] Petersson G.A., Al-Laham M.A. (1991). A complete basis set model chemistry. II. Open-shell systems and the total energies of the first-row atoms. J. Chem. Phys..

[28] Hay P.J., Wadt W.R. (1985). Ab initio effective core potentials for molecular calculations. Potentials for the transition metal atoms Sc to Hg. J. Chem. Phys..

[29] Wadt W.R., Hay P.J. (1985). Ab initio effective core potentials for molecular calculations. Potentials for main group elements Na to Bi. J. Chem. Phys..

[30] Hay P.J., Wadt W.R. (1985). Ab initio effective core potentials for molecular calculations. Potentials for K to Au including the outermost core orbitale. J. Chem. Phys..

[31] Cossi M., Rega N., Scalmani G., Barone V. (2003). Energies, Structures, and Electronic Properties of Molecules in Solution with the C-PCM Solvation Model. J. Comput. Chem..

[32] Ullah R., Zheng Y. (2016). Raman spectroscopy of “Bisphenol A”. J. Mol. Struct..

[33] Ullah R., Wang X. (2019). Raman spectroscopy of Bisphenol ‘S’ and its analogy with Bisphenol ‘A’ uncovered with a dimensionality reduction technique. J. Mol. Struct..

[34] Barnett S.M., Vlckova B., Butler I.S., Kanigan T.S. (1994). Surface-Enhanced Raman Scattering Spectroscopic Study of 17.alpha.-Ethinylestradiol on Silver Colloid and in Glass-Deposited Ag-17.alpha.-Ethinylestradiol Film. Anal. Chem..

[35] Han X.X., Pienpinijtham P., Zhao B., Ozaki Y. (2011). Coupling Reaction-Based Ultrasensitive Detection of Phenolic Estrogens Using Surface-Enhanced Resonance Raman Scattering. Anal. Chem..

[36] De Bleye C., Dumont E., Hubert C., Sacré P.Y., Netchacovitch L., Chavez P.F., Hubert P., Ziemons E. (2015). A simple approach for ultrasensitive detection of bisphenols by multiplexed surface-enhanced Raman scattering. Anal. Chim. Acta.

[37] Guerrini L., Garcia-Ramos J.V., Domingo C., Sanchez-Cortes S. (2006). Functionalization of Ag nanoparticles with dithiocarbamate calix[4]arene as an effective supramolecular host for the surface-enhanced Raman scattering detection of polycyclic aromatic hydrocarbons. Langmuir.

[38] Guerrini L., Garcia-Ramos J.V., Domingo C., Sanchez-Cortes S. (2009). Self-assembly of a dithiocarbamate calix[4]arene on Ag nanoparticles and its application in the fabrication of surface-enhanced Raman scattering based nanosensors. Phys. Chem. Chem. Phys..

[39] Lai H., Xu F., Zhang Y., Wang L. (2018). Recent progress on graphene-based substrates for surface-enhanced Raman scattering applications. J. Mater. Chem. B.

[40] Xie Y., Wang X., Han X., Xue X., Ji W., Qi Z., Liu J., Zhao B., Ozaki Y. (2010). Sensing of polycyclic aromatic hydrocarbons with cyclodextrin inclusion complexes on silver nanoparticles by surface-enhanced Raman scattering. Analyst.

[41] Fang C., Bandaru N.M., Ellis A.V., Voelcker N.H. (2013). Beta-cyclodextrin decorated nanostructured SERS substrates facilitate selective detection of endocrine disruptor chemicals. Biosens. Bioelectron..

[42] Lofrumento C., Platania E., Ricci M., Becucci M., Castellucci E.M. (2016). SERS Spectra of Alizarin Anion-Agn (n = 2, 4, 14) Systems: TDDFT Calculation and Comparison with Experiment. J. Phys. Chem. C.

[43] Ricci M., Lofrumento C., Becucci M., Castellucci E.M. (2018). The Raman and SERS spectra of indigo and indigo-Ag2 complex: DFT calculation and comparison with experiment. Spectrochim. Acta Part A Mol. Biomol. Spectrosc..

[44] López-Tocón I., Valdivia S., Soto J., Otero J.C., Muniz-Miranda F., Menziani M.C., Muniz-Miranda M. (2019). A DFT approach to the surface-enhanced Raman scattering of 4-cyanopyridine adsorbed on silver nanoparticles. Nanomaterials.

[45] Agarwal N.R., Lucotti A., Tommasini M., Neri F., Trusso S., Ossi P.M. (2016). SERS detection and DFT calculation of 2-naphthalene thiol adsorbed on Ag and Au probes. Sens. Actuators B Chem..

[46] Varsanyi G. (1969). Vibrational Spectra of Benzene Derivatives.

[47] Schaftenaar G., Noordik J.H. (2000). Molden: A pre- and post-processing program for molecular and electronic structures. J. Comput. Aided Mol. Des..

