# Supplementary material for: Surface-Enhanced Raman Spectroscopy for Bisphenols Detection: Toward a Better Understanding of the Analyte–Nanosystem Interactions
Source: Nanomaterials (Basel). 2021 Mar 30;11(4):881. doi: 10.3390/nano11040881 (PMC8067303; doi:10.3390/nano11040881)
Supplement: Supplementary file 1 [file nanomaterials-11-00881-s001.pdf]

Supplementary Materials

Surface-Enhanced Raman Spectroscopy for Bisphenols Detection: Toward a Better Understanding of the Analyte–Nanosystem Interactions

Eleonora Roschi [1], Cristina Gellini [1], Marilena Ricci [1], Santiago Sanchez-Cortes [2], Claudia Focardi [3], Bruno Neri [4], Juan Carlos Otero [5], Isabel López-Tocón [5], Giulietta Smulevich [1,6,*] and Maurizio Becucci [1,7,*]

1 Dipartimento di Chimica "Ugo Schiff", Università di Firenze, I-50019 Sesto Fiorentino (Fi), Italy; eleonora.roschi@gmail.com (E.R.); cristina.gellini@unifi.it (C.G.); marilena.ricci@unifi.it (M.R.)

2 Instituto de Estructura de la Materia, IEM-CSIC, Serrano, 121. E-28006 Madrid, Spain; s.sanchez.cortes@csic.es

3 Istituto Zooprofilattico Sperimentale delle Regioni Lazio e Toscana, I-50010, San Martino alla Palma (FI), Italy; claudia.focardi@izslt.it

4 Istituto Zooprofilattico Sperimentale delle Regioni Lazio e Toscana, I-00178, Roma, Italy; bruno.neri@izslt.it

5 Andalucía Tech, Unidad Asociada IEM-CSIC, Departamento de Química Física, Facultad de Ciencias, Universidad de Málaga, E-29071 Málaga, Spain; jc_otero@uma.es (J.C.O.); tocon@uma.es (I.L.-T.)

6 INSTM Research Unit of Firenze, I-50019 Sesto Fiorentino (Fi), Italy

7 LENS, European Laboratory for Non Linear Spectroscopy, I-50019 Sesto Fiorentino (Fi), Italy

* Correspondence: giulietta.smulevich@unifi.it (G.S.); maurizio.becucci@unifi.it (M.B.)

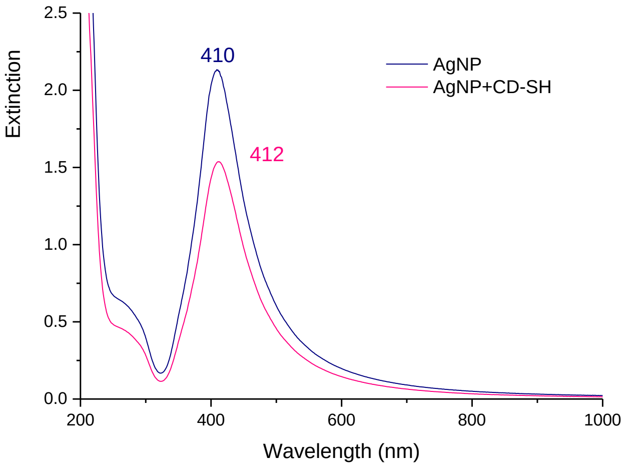

Figure S1. Localized surface plasmon resonance (LSPR) band of silver nanoparticles from synthesis (black line) and after functionalization with CD-SH (red line). Optical pathlength 2 mm

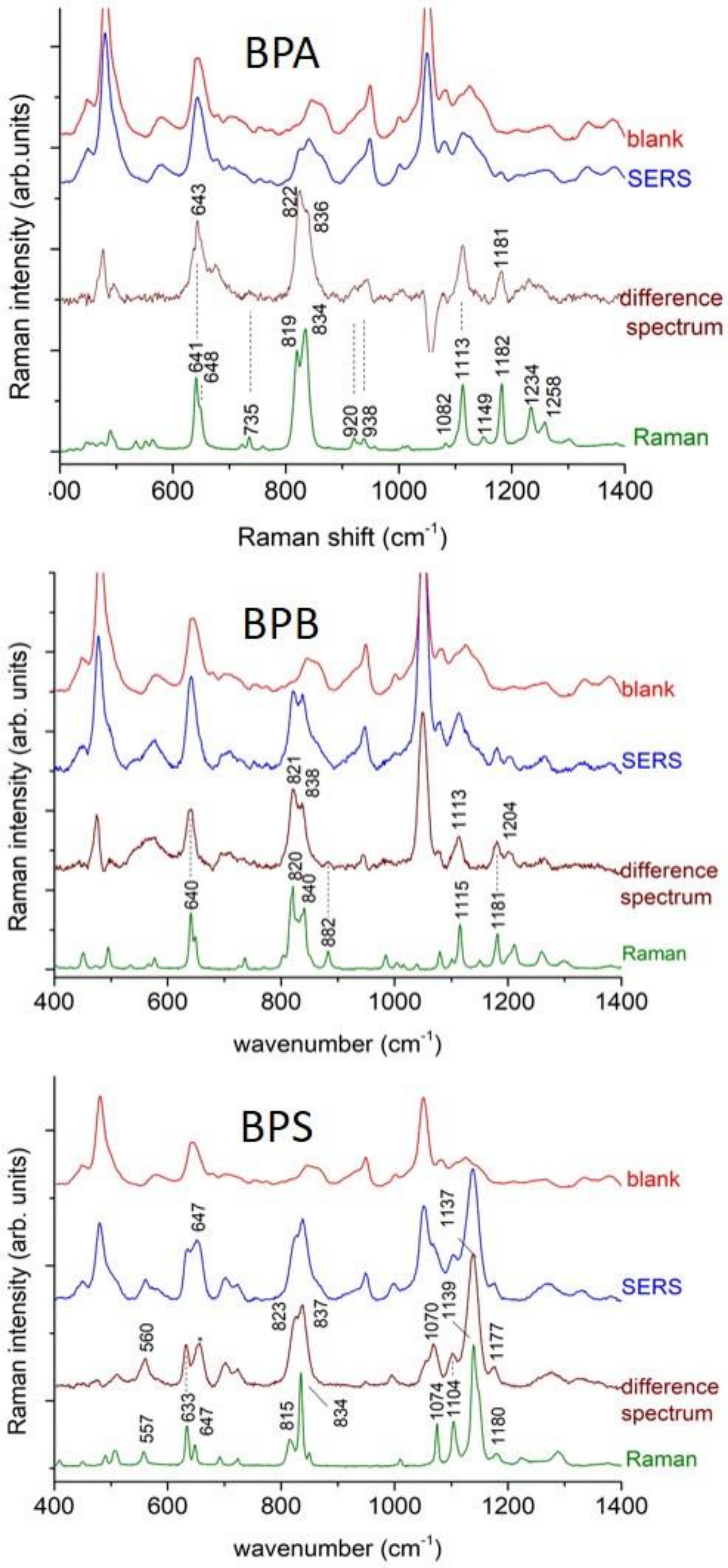

Figure S2. The background-subtracted SERS spectra of the different BPs compared to the corresponding Raman spectra. Background subtracted spectra are difference spectra between the experimental SERS spectra of the sample and the corresponding blank SERS spectra. The major CD Raman bands are not always perfectly removed by the procedure and appear in the difference spectrum either as residual negative or positive peaks.

Table S1. Assignment of the main Raman and SERS bands (cm^{-1}) of BPA, BPB and BPS previously reported [17,18].

BPA		BPB		BPS		Assignment [31-32]
Raman	SERS	Raman	SERS	Raman	SERS	
489(w)	495[1]	494(w)	474[1]			γ(CO)[31],
				557(w)	560(w)	γ(OCOS)[32]
641(ms)	643[2]	640 (ms)	641[2]	633(m)	633(m)[2]	δ(CCC)r[31]
				647(w)	647(m)[2]	ν(SC)[32]
648(sh)		648(sh)				ν(CC)[31]
735(w)		736(w)				τ(ring)[31], γ(CO)[31]
819(s)	822(s)	820(s)	821(s)	815(w)	823(ms)	γ(CH)[31],τ(HCCC)[32]
834(s)	836(s)	840(s)	838(s)	834(s)	837(s)	δ(CCC)r[32],γ(CH)[31]
		882(w)				
		984(w)				
920(w)	922 (w)					ν(CC)[31]
938(w)	938(w)					ν(CC)[31],ω(CCH)r[31]
1083(vw)		1079(w)		1074(m)	1074(sh)	ν(CC)[31]
1113(m)	1113(m)	1115(m)	1113(m)	1104(m)	1104(w)	ν(CC)[32],ω(CCH)r[31]
				1139(s)	1137(s)	ν(SO)[32]
1149(vw)						ν(CC)[31]
1182(m)	1181(w)	1181(m)	1181(w)	1179 (w)	1177(w)	ν(CC)[32],ω(CCH)r[31]
		1211(w)	1204(w)			
1234(w)						ν(CO)[31]
1257(w)		1259(w)				ν(CC)[31]

[1] overlapped with the band at 480 cm^{-1} of CD-SH; [2] overlapped with the band at 643 cm^{-1} of CD-SH

sh, shoulder; s, strong; ms, medium strong; m, medium; w, weak.

ν, stretching; δ, in-plane deformation; τ, torsion deformation; r, rocking; sym, symmetric motion; asym, asymmetric motion.

Table S2. Main Raman bands (cm^{-1}) of BPA, BPB and BPS: Comparison between experimental and B3LYP/6-31G* calculated results in this work.

Experimental[1]			B3LYP/6-31G*			
BPA	BPB	BPS	BPA	BPB	BPS	Assignment[2]
489(w)	494(w)		543	551		δCCCskeletal
		557(w)			560	rCSC + rSO
641(ms)	640(ms)	633(m)	645	636	638	6b;δring + νCring-C-Cring
648(sh)	648(sh)	647(w)	657	658	648	6a;δring
735(w)	736(w)		744	748		4;τring
819(s)	820(s)	815(w)	847	848	846	1;νring
834(s)	840(s)	834(s)	857	868	853	1;νring + νCring-C-Cring
920(w)			950			rCH_3 + νCCH_3
938(w)			958			rCH_3 + νCCH_3
1083(vw)	1079(w)	1074(m)	1110	1110	1110	δCHring + νCCring (+rCH_3 in BPA,B or νasymSO in BPS)
1113(m)	1115(m)	1104(m)	1133	1139	1138	δCHring + νCCring
		1139(s)			1136	νsymSO + δCHring
1149(vw)			1178			δCHring + νCCH_3
1182(m)	1181(m)	1179(w)	1210	1215		δCOH + δCHring
	1211(w)			1235		δCHring + νCring-C-Cring
1234(w)			1268			νCring-C-Cring + $rCCH_3$
1257(w)	1259(w)		1308	1310		νCO + νCCring + δCHring

[1] sh, shoulder; s, strong; ms, medium strong; m, medium; w, weak.

[2] ν, stretching; δ, in-plane deformation; τ, torsion deformation; r, rocking; sym, symmetric motion; asym, asymmetric motion. Wilson´s nomenclature for the benzene-like normal modes, 6a, 6b, 1, and 4 [45]. Vibrational modes visualized by using MOLDEN program[46].

.

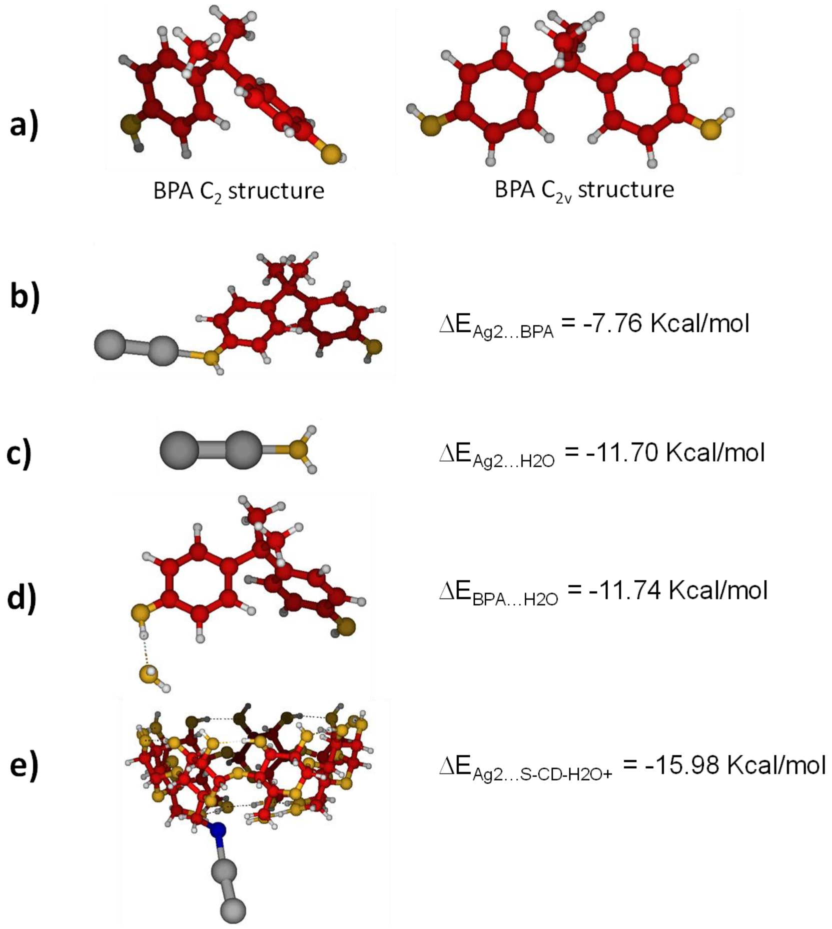

Figure S3. (a) B3LYP/6-31G* optimized geometries of BPA and (b-e) B3LYP/LanL2DZ interaction energies of different complexes formed between BPA, Ag_2, H_2O and CD-SH.

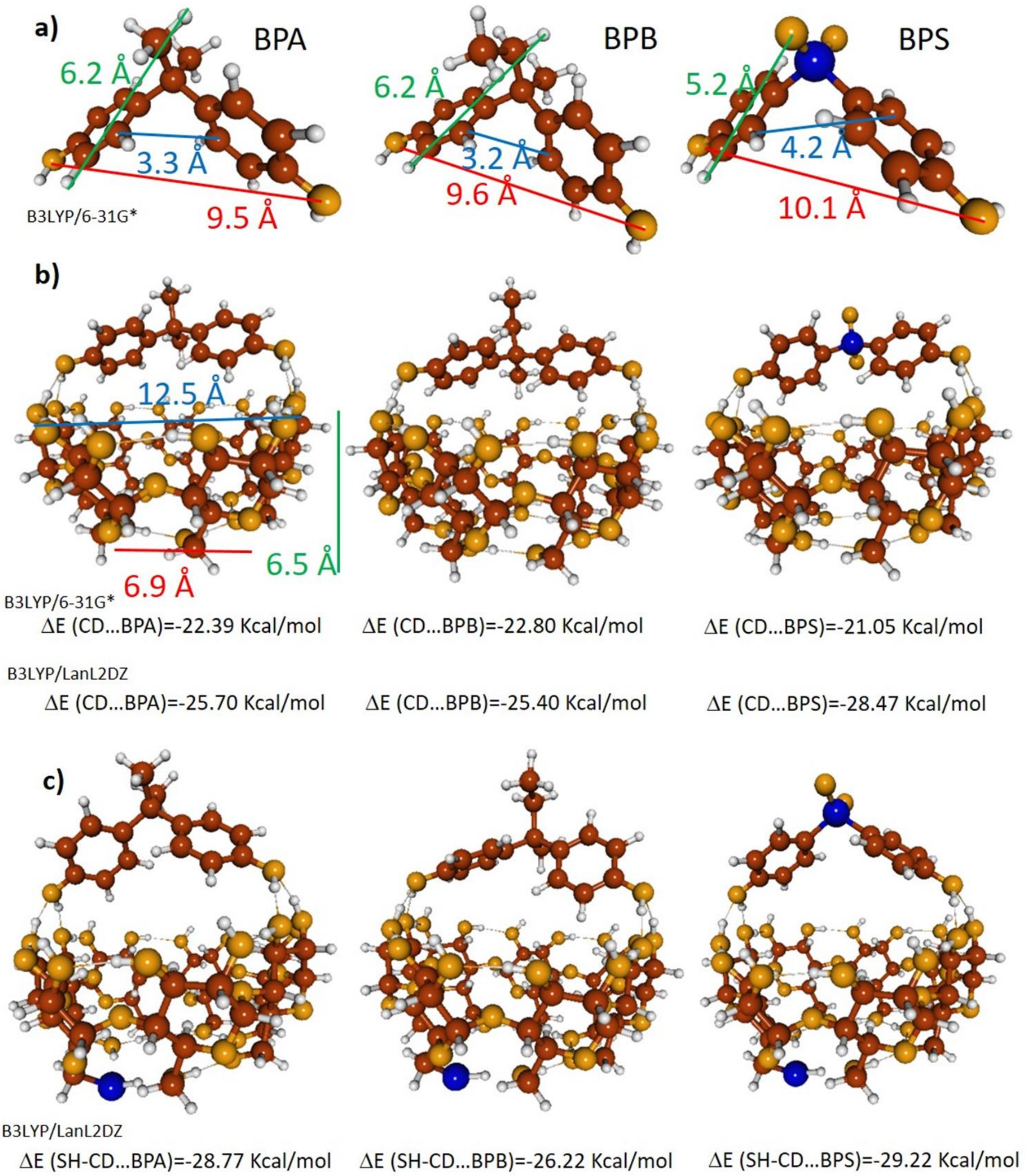

Figure S4. (a) and (b) B3LYP/6-31G* optimized geometries of isolated BPs and of the three complexes formed between BPs and CD with their corresponding calculated interaction energies (B3LYP/6-31G* and B3LYP/LanL2DZ). (c) B3LYP/LanL2DZ optimized geometries and calculated interaction energies of the three complexes formed between BPs and SH-CD.

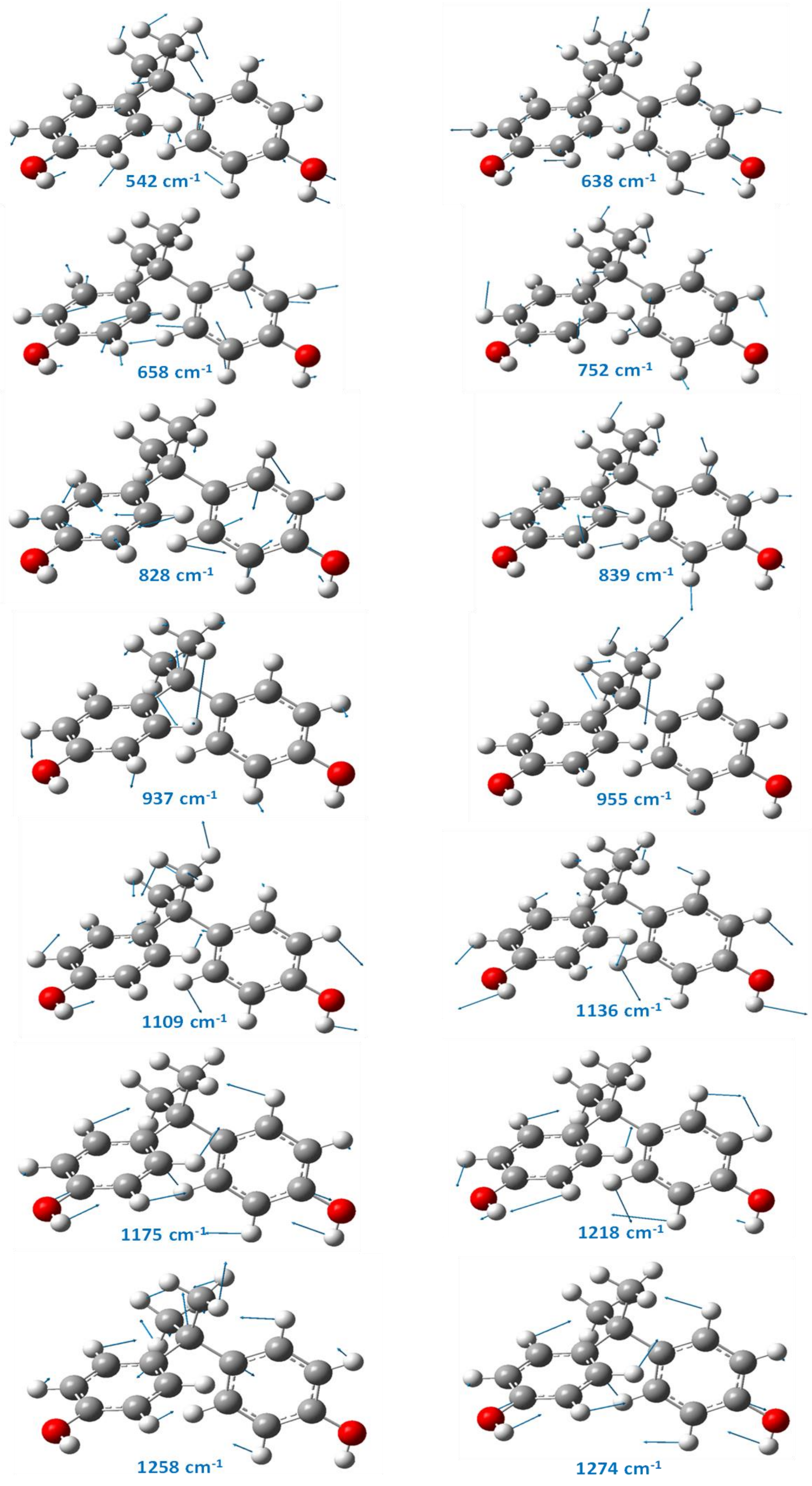

Figure S5. B3LYP/LanL2DZ calculated atomic displacements of the main normal modes of BPA.

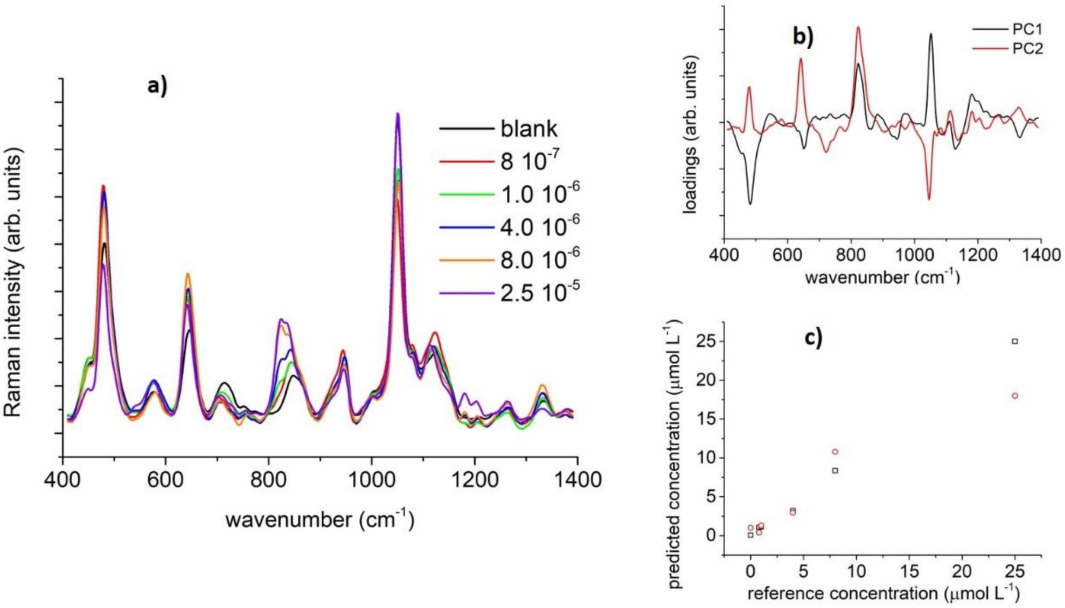

Figure S6. Relevant SERS data for BPB and they multivariate analysis. a) BPB SERS spectra as a function of the nominal BPB molar concentration (SNV data transformation and smoothing applied). b) loadings for the first two components in the PCA analysis on the BPB SERS data (full spectrum). PC1 and PC2 are carrying the 53 and 36 percent of the spectral variance, respectively. c) predicted (black square) and validated (red circle) concentration of BPB in model samples vs. reference analytical concentration as obtained by the partial least square (PLS) regression analysis (4 factors model) on SERS data (full spectrum).

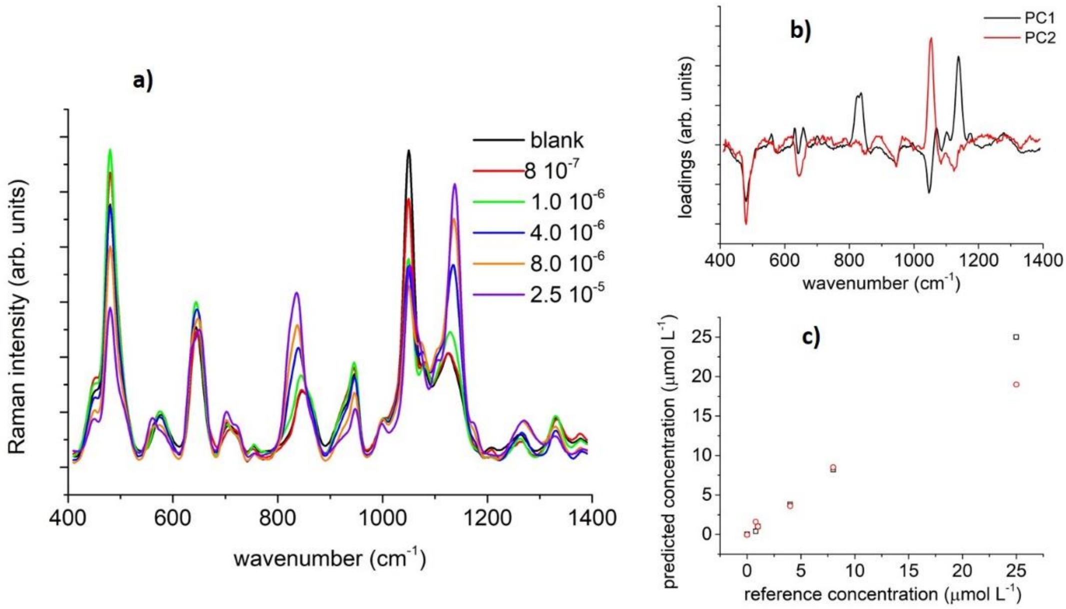

Figure S7. Relevant SERS data for BPS and they multivariate analysis. a) BPS SERS spectra as a function of the nominal BPB molar concentration (SNV data transformation and smoothing applied). b) loadings for the first two components in the PCA analysis on the BPS SERS data (full spectrum). PC1 and PC2 are carrying the 85 and 12 percent of the spectral variance, respectively. c) predicted (black square) and validated (red circle) concentration of BPS in model samples vs. reference analytical concentration as obtained by the partial least square (PLS) regression analysis (4 factors model) on SERS data (full spectrum).
